# Differences in heart rate responses to upright posture are associated with variations in the high-frequency power of heart rate variability

**DOI:** 10.1152/ajpheart.00567.2023

**Published:** 2023-12-22

**Authors:** Heidi Bouquin, Jenni K. Koskela, Antti Tikkakoski, Milja Honkonen, Timo P. Hiltunen, Jukka T. Mustonen, Ilkka H. Pörsti

**Affiliations:** ^1^Faculty of Medicine and Health Technology, Tampere University, Tampere, Finland; ^2^Department of Internal Medicine, Tampere University Hospital, Tampere, Finland; ^3^Department of Clinical Physiology and Nuclear Medicine, Tampere University Hospital, Tampere, Finland; ^4^Research Program for Clinical and Molecular Metabolism, Faculty of Medicine, University of Helsinki, Helsinki, Finland; ^5^Faculty of Medicine, University of Helsinki and Helsinki University Hospital, Helsinki, Finland; ^6^Finnish Cardiovascular Research Center Tampere, Tampere University, Tampere, Finland

**Keywords:** head-up tilt, heart rate, phenotype

## Abstract

High resting heart rate is a cardiovascular risk factor, but limited data exist on the underlying hemodynamics and reproducibility of supine-to-upright increase in heart rate. We recorded noninvasive hemodynamics in 574 volunteers [age, 44.9 yr; body mass index (BMI), 26.4 kg/m^2^; 49% male] during passive head-up tilt (HUT) using whole body impedance cardiography and radial artery tonometry. Heart rate regulation was evaluated using heart rate variability (HRV) analyses. Comparisons were made between quartiles of supine-to-upright heart rate changes, in which heart rate at rest ranged 62.6–64.8 beats/min (*P* = 0.285). The average upright increases in heart rate in the *quartiles 1–4* were 4.7, 9.9, 13.5, and 21.0 beats/min, respectively (*P* < 0.0001). No differences were observed in the low-frequency power of HRV, whether in the supine or upright position, or in the high-frequency power of HRV in the supine position. Upright high-frequency power of HRV was highest in *quartile 1* with lowest upright heart rate and lowest in *quartile 4* with highest upright heart rate. Mean systolic blood pressure before and during HUT (126 vs. 108 mmHg) and the increase in systemic vascular resistance during HUT (650 vs. 173 dyn·s/cm^5^/m^2^) were highest in *quartile 1* and lowest in *quartile 4*. The increases in heart rate during HUT on three separate occasions several weeks apart were highly reproducible (*r* = 0.682) among 215 participants. To conclude, supine-to-upright increase in heart rate is a reproducible phenotype with underlying differences in the modulation of cardiac parasympathetic tone and systemic vascular resistance. As heart rate at rest influences prognosis, future research should elucidate the prognostic significance of these phenotypic differences.

**NEW & NOTEWORTHY** Subjects with similar supine heart rates are characterized by variable increases in heart rate during upright posture. Individual heart rate increases in response to upright posture are highly reproducible as hemodynamic phenotypes and present underlying differences in the modulation of cardiac parasympathetic tone and systemic vascular resistance. These results indicate that resting heart rate obtained in the supine position alone is not an optimal means of classifying people into groups with differences in cardiovascular function.

## INTRODUCTION

The evaluation of cardiovascular status is usually carried out by recording blood pressure (BP) and heart rate (HR) in the seated position (1). Additional BP and HR measurements while standing are recommended in the elderly, in patients with neurodegenerative disease or diabetes, and when postural hypotension is suspected (1). However, even in the absence of cardiovascular disease, the changes in systolic BP during orthostatic challenge are not uniform (2). In 1,207 hypertensive subjects, exaggerated systolic BP response to standing, also characterized by higher supine-to-standing increase in HR, was a predictor of adverse cardiovascular events during 17 years of follow-up (3).

Epidemiological studies have linked elevated resting HR with higher cardiovascular morbidity (4, 5). Higher resting HR is an indicator of increased cardiovascular event rate in both sexes, but the effect is more pronounced in men (5–7). In the general population, higher resting HR predicts all-cause mortality, independent of classic cardiovascular risk factors (8). Higher resting HR is characterized by a hyperdynamic hemodynamic profile and higher HR also present in the upright position (9). Analyses of heart rate variability (HRV) have suggested that higher resting HR is related to lower high-frequency (HF) power of HRV in both supine and upright positions, suggesting a lower parasympathetic influence on HR modulation (10). Sympathetic overactivity is a putative link between elevated HR and cardiovascular events (11, 12), and this may explain the significance of resting HR as a prognostic factor (13).

Although great proportions of our active hours are spent in the upright position, detailed information about upright hemodynamics remains rather scarce. While standing, blood pools into the lower extremities, decreasing venous return and cardiac output (2). Subsequently, BP is maintained by an increase in sympathetic tone, decrease in parasympathetic tone, and an elevation in HR and systemic vascular resistance (14–16). However, phenotypic differences exist in the underlying changes of systemic vascular resistance and cardiac output during upright posture (17).

Passive head-up tilt (HUT) and active orthostatic challenge have been predominantly used to study the regulation of HR and other hemodynamic variables in patients with a history of syncope (18). Here, we tested the hypothesis of whether phenotypic differences exist in the HR responses during passive HUT in a group of 574 individuals of Finnish descent. Furthermore, we investigated whether these differences are reproducible among a smaller group comprising 215 subjects.

## MATERIALS AND METHODS

### Study Population

This investigation is part of the DYNAMIC study (EudraCT 2006-002065-39, Clinical Trials registration NCT01742702), focusing on noninvasive hemodynamics. The study was approved by the Ethics Committee of Tampere University Hospital (Study Code R06086M) and conforms with the principles of the Declaration of Helsinki. Written informed consent was received from all participants. Recruitment was through several channels: announcements among occupational health-care providers, Tampere University, Tampere University Hospital, and Varala Sports Institute in Tampere, and two announcements were published in a local newspaper (19, 20). Participants were enrolled to the study in the order the research nurses received their contact information. To this day, 1,442 subjects have participated.

The participants in this study consisted of 574 Finnish subjects (49% male, aged 19–73 yr) who had available data from noninvasive hemodynamic recordings and HRV analyses and did not meet any exclusion criteria. Altogether, 98% of the participants were without medications directly affecting cardiovascular function. Blood tests were drawn after ∼12-h fasting, and morning urine samples were collected. Kidney diseases were ruled out by plasma creatinine and urine dipstick analyses. Other exclusion criteria were any acute illness, body mass index (BMI) > 40, missing key hemodynamic data, history of cardiovascular disease, absence of sinus rhythm, chronic liver disease, secondary hypertension, psychiatric illness other than mild depression or anxiety, and alcohol or substance abuse. Four patients with type 2 diabetes with good glycemic control were included.

The average seated office HR was 67 beats/min, and the average office BP was 138/88 mmHg, measured according to the European Society of Hypertension guidelines by a physician (1). Lifestyle habits were registered, including data on diet, number of ≥30-min bouts of exercise/wk, consumption of alcohol, and smoking status with cigarette consumption. Personal and family medical histories were noted.

Medications used by the participants were female hormones (*n* = 73), vitamin D (*n* = 41), other dietary supplements (vitamins, minerals, omega-3 fatty acids) (*n* = 45), antidepressants (*n* = 34), hormonal intrauterine devices (*n* = 23), asthma medication (*n* = 20), thyroxine (*n* = 18), statins (*n* = 17), inhaled glucocorticoids (*n* = 17), antihistamines (*n* = 16), proton pump inhibitors (*n* = 12), low-dose acetylsalicylic acid (*n* = 11), antirheumatics (*n* = 7), nonsteroidal anti-inflammatory drugs (*n* = 7), topically applied estrogens (*n* = 6), glaucoma medication (*n* = 6; timolol by 5), inhaled β_2_-mimetics (*n* = 5), metformin (*n* = 4), gabapentin or pregabalin (*n* = 4), hypnotics (*n* = 4), allopurinol (*n* = 3), analgesics (*n* = 3), anxiolytics (*n* = 3), dipeptidyl peptidase-4 inhibitors (*n* = 2), anticoagulants (*n* = 2), antiepileptics (*n* = 2), intramuscular vitamin B_12_ (*n* = 2), muscle relaxants (*n* = 2), varenicline (*n* = 2), ezetimibe (*n* = 1), sulphonylurea (*n* = 1), insulin (*n* = 1), anticholinergic (*n* = 1), antiestrogen (*n* = 1), bisphosphonate (*n* = 1), cholestyramine (*n* = 1), montelukast (*n* = 1), and retinoid (*n* = 1). Three of the medicines used by subjects had possible cardiac effects: β_2_-mimetics, timolol, and anticholinergics. The β_2_-mimetic and timolol users were evenly distributed within the quartiles, and only one subject used anticholinergic medication, and all these subjects were kept in the study.

### Experimental Protocol

The hemodynamic measurements were performed by a trained research nurse in a quiet, temperature-controlled laboratory as previously described (21, 22). In brief, while on the tilt table, the impedance cardiography electrodes were placed on body surface, the tonometric sensor for pulse wave analysis was placed on the radial pulsation of the left wrist, and the oscillometric brachial cuff for blood pressure (BP) calibration was attached to the right upper arm (21, 22). The left arm was abducted to 90° in a support in which arm, sensor, and probes were at the heart level whether in supine or upright position. An introductory HUT preceded the actual measurement (21). Then hemodynamic recordings were captured continuously for 5 min in supine position, followed by 5 min of passive HUT at ≥60°. The recorded mean values of each 1-min period were calculated and used in the analyses. The subjects were instructed to abstain from smoking, using caffeinated products, and consuming heavy meals for ≥4 h and alcoholic beverages for ≥24 h before the protocol.

### Pulse Wave Analysis and Whole Body Impedance Cardiography

A tonometric sensor on the radial artery continuously captured the pulse waveform (Colin BP-508T, Colin Medical Instruments, San Antonio, TX), which was then transferred to the SpygmoCor PWMx monitoring system (AtCor Medical, New South Wales, Australia). The system estimated aortic BP continuously (23), and average values for each minute were calculated.

Changes in body electrical impedance were recorded using whole body impedance cardiography (CircMon, JR Medical, Tallinn, Estonia) to determine beat-to-beat HR, stroke volume, cardiac output, and pulse wave velocity (PWV). Systemic vascular resistance and left cardiac work were calculated from the BP signal of the tonometric sensor, and the cardiac output was evaluated using the CircMon device (22). Stroke volume, cardiac output, systemic vascular resistance, and left cardiac work were presented as indexes adjusted to body surface area [stroke index (SI), cardiac index (CI), systemic vascular resistance index (SVRI), and left cardiac work index (LCWI), respectively]. LCWI was calculated as follows: 0.0143·(mean aortic pressure − pulmonary artery occlusion pressure)·CI (22, 24). Assumed mean normal values of central venous (3 mmHg) and pulmonary arterial occlusion (6 mmHg) pressures were used in calculations (22, 25, 26).

### Heart Rate Variability Analyses

Modulation of heart rate was assessed using HRV analyses from single-channel electrocardiogram recordings of the CircMon system (sampling rate, 200 Hz). Normal R-R intervals were recognized and beats that varied >20% from previous values were regarded as ectopic. The cubic spline interpolation method was used for dealing with artifacts. MATLAB software (MathWorks, Natick, MA) was used, and the fast Fourier transformation method was used to calculate the frequency domain variables: *1*) power in low-frequency (LF) range (0.04–0.15 Hz), *2*) power in HF range (0.15–0.40 Hz), and *3*) LF:HF ratio (27).

### Laboratory Analyses

Blood and urine samples were collected after ∼12 h of fasting and analyzed in Fimlab Laboratories, Tampere, Finland (www.fimlab.fi). Quantitative insulin sensitivity check index (QUICKI) was calculated from fasting plasma glucose and insulin (28). Plasma C-reactive protein (CRP), sodium, potassium, calcium, phosphate, glucose, creatinine, triglyceride, and total, high-density, and low-density lipoprotein cholesterol concentrations were determined using Cobas Integra 700/800 chemistry analyzer (Roche Diagnostics, Basel, Switzerland). Blood cell count was analyzed using ADVIA 2120 hematology systems (Siemens, Munich, Germany). Insulin concentrations were determined using electrochemiluminescence immunoassay Cobas e411 (Roche Diagnostics, Basel, Switzerland).

### Reproducibility

The reproducibility of the HR phenotype in three separate HUT tests was tested in 215 participants. No medications with direct influences on cardiovascular function were used by the participants. Median time differences between recordings were as follows: 61 days between the first and the second recording and 29 days between the second and the third recording.

These 215 subjects were examined in sex-specific quartiles according to the HR change during the first HUT. The quartiles presented no differences in the prevalence of smokers, alcohol consumption, or in the same laboratory analysis results performed on the main study cohort (not shown). The quartile ages [standard deviations (SDs)] were 47.8 (10.3), 47.2 (9.2), 45.3 (8.6), and 41.6 (10.7) yr; the fourth quartile with the highest HR increase in HUT was younger than the first and the second quartiles (*P* < 0.05), which corresponds to the results of the main study.

### Statistics

Mean supine (SD) HR in women was 64.5 (9.4) and in men was 62.6 (9.8) beats/min (*P* = 0.015), whereas the mean change in HR in response to HUT in women was 11.4 (6.3) and in men was 13.2 (6.8) beats/min (*P* = 0.001), respectively. To eliminate sex-related confounding (16, 22, 29), men and women were divided separately into quartiles (*Q1*–*Q4*) of the supine-to-upright change in HR. These HR quartiles, which were separately formed for both sexes, were used as the study groups of interest for the examination of all other hemodynamic variables.

Independent sample *t* test and one-way analysis of variance (ANOVA), supported by the Bonferroni post hoc test for normally distributed variables, and Kruskal–Wallis test, supported by the post hoc Dunn test for variables with skewed distribution, were applied to examine the clinical characteristics and laboratory values. HRV statistics were analyzed from logarithmically transformed values recorded during the whole 5-min periods in supine and upright postures.

The mean values of each 1-min period of the hemodynamic variables were calculated. ANOVA for repeated measures was applied to compare hemodynamic variables: all values from the 5 min in supine posture and values from the 3rd, 4th, and 5th min in upright posture were included in the analyses, as the upright responses were only stable during the last 3 min. The change in response to HUT was calculated as the difference between the mean value of the last 3 min in supine posture and the last 3 min in upright posture. The analyses were adjusted for age, BMI, and fasting plasma glucose, as appropriate. PWV was given as the mean of all recorded values during 5 min in the supine position, and it was additionally adjusted for mean aortic pressure (30).

Variables that were normally distributed are reported as means (SD) or as means (SE). Nonnormally distributed variables are reported as medians [25th–75th percentiles]. Pearson correlations (*r*_P_) were calculated when appropriate. The Bonferroni correction was applied in all post hoc analyses, and *P* < 0.05 was considered significant. IBM Statistics SPSS version 28 was used (IBM SPSS, Armonk, NY).

## RESULTS

### Study Population and Results of Blood and Plasma Analyses

The demographics and characteristics of the quartiles (*Q1*–*Q4*) of the change in HR during HUT are shown in Table 1 (smallest increase in HR in *Q1* and largest in *Q4*). Subjects in *Q4* were the youngest, whereas age was also lower in *Q3* than in *Q1*. Mean study population body mass index (BMI) was 26.5 (4.5) kg/m^2^. There were no differences in weight, but subjects in *Q4* were taller than in *Q2*, and BMI was lower in *Q4* than in *Q2*. The prevalence of current smokers and alcohol consumption was not different between the quartiles.

**Table 1.** Demographics and clinical characteristics in quartiles of supine-to-upright change in heart rate divided separately for sexes

	*Quartile 1*	*Quartile 2*	*Quartile 3*	*Quartile 4*	*P* Value
*n*	142	140	154	138	
Males/females, %	49/51	52/48	48/52	49/51	0.903
Age, yr	49.5 (11.1)	46.4 (11.0)	44.2 (11.4)*	39.3 (11.6)*†‡	<0.001
Weight, kg	79.7 (14.6)	79.8 (15.4)	79.3 (14.5)	78.7 (16.5)	0.935
Height, cm	172.2 (8.6)	171.8 (8.9)	173.5 (8.9)	175.0 (10.0)†	0.014
Body mass index, kg/m^2^	26.8 (4.1)	27.0 (4.2)	26.3 (4.1)	25.6 (4.4)†	0.030
Current smokers, %	8.5	14.3	13.6	18.8	0.093
Alcohol consumption, standard drinks/wk	2 [1–7]	3 [1/6]	2 [1/5]	3 [1/6]	0.341
Seated office measurements					
Heart rate, beats/min	65 (9)	68 (11)	67 (9)	68 (10)	0.114
Systolic blood pressure, mmHg	145 (20)	141 (19)	138 (18)*	130 (16)*†‡	<0.001
Diastolic blood pressure, mmHg	90 (11)	90 (12)	88 (12)	85 (11)*†	0.001
Pulse wave velocity, m/s	8.2 (1.2)	8.4 (1.2)	8.0 (1.2)	8.2 (1.3)	0.055

Values are means (SD) or medians [25th–75th percentiles]; *n*, number of participants. Pulse wave velocity analyses were adjusted for age, body mass index, fasting plasma glucose concentration, and mean aortic pressure. *Quartile 1* had lowest and *quartile 4* highest heart rate change during head-up tilt. *P < 0.05 vs. *quartile 1*; †*P* < 0.05 vs. *quartile 2*; ‡*P* < 0.05 vs. *quartile 3*.

Among all participants, average seated office HR was 67 beats/min and average office BP 138/88 mmHg. Seated office HR was similar in the quartiles (Table 1). Seated office systolic BP was lowest in *Q4* and was also lower in *Q3* than in *Q1*. Diastolic office BP was lower in *Q4* than in *Q1* and *Q2*. Aortic-to-popliteal pulse wave velocities were not different among the quartiles (Table 1).

Blood hemoglobin showed minor clinically insignificant differences, whereas fasting plasma C-reactive protein (CRP), electrolytes, and creatinine were similar in all quartiles (Table 2). Total cholesterol was lower in *Q4* than in *Q2*, but no other differences were observed in plasma lipids. Fasting plasma glucose was lower in *Q4* than in *Q1*, whereas no significant differences in plasma insulin concentrations were observed. Insulin sensitivity, as judged by quantitative insulin sensitivity check index (QUICKI), was similar in all quartiles (Table 2).

**Table 2.** Laboratory results of blood and plasma samples in quartiles of supine-to-upright change in heart rate divided separately for sexes

	*Quartile 1*	*Quartile 2*	*Quartile 3*	*Quartile 4*	*P* Value
*n*	142	140	154	138	
Blood hemoglobin, g/L	144.0 (11.0)	146.0 (13.0)	143.0 (13.0)	145.0 (12.0)	0.042
C-reactive protein, mg/L	0.9 [0.5–1.5]	0.9 [0.5–1.9]	0.5 [0.5–1.4]	0.7 [0.5–1.9]	0.626
Potassium, mmol/L	3.7 (0.3)	3.8 (0.3)	3.8 (0.3)	3.8 (0.2)	0.680
Sodium, mmol/L	141 (2)	140 (2)	141 (2)	141 (2)	0.779
Calcium, mmol/L	2.3 (0.1)	2.3 (0.1)	2.3 (0.1)	2.3 (0.1)	0.828
Phosphate, mmol/L	0.9 (0.2)	1.0 (0.2)	1.0 (0.2)	1.0 (0.2)	0.273
Creatinine, μmol/L	74 (15)	75 (13)	75 (14)	74 (13)	0.354
Total cholesterol, mmol/L	5.1 [4.6–5.8]	5.4 [4.4–6.1]	5.1 [4.4–5.7]	5.0 [4.3–5.6]†	0.020
HDL cholesterol, mmol/L	1.6 [1.3–1.9]	1.6 [1.3–1.9]	1.5 [1.3–1.9]	1.5 [1.3–1.9]	0.193
LDL cholesterol, mmol/L	3.1 [2.4–3.8]	3.2 [2.3–3.9]	2.9 [2.3–3.5]	2.8 [2.2–3.4]	0.145
Triglycerides, mmol/L	1.1 [0.8–1.3]	1.0 [0.8–1.5]	1.0 [0.8–1.5]	1.0 [0.7–1.4]	0.465
Fasting glucose, mmol/L	5.5 (0.7)	5.5 (0.5)	5.5 (0.6)	5.3 (0.5)*	0.006
Insulin, mU/L	8.0 (5.0)	8.8 (7.1)	8.5 (5.9)	8.2 (6.0)	0.706
QUICKI	0.356 (0.04)	0.356 (0.04)	0.358 (0.05)	0.362 (0.05)	0.686

Values are means (SD) or medians [25th–75th percentiles]; *n*, number of participants. HDL, high-density lipoprotein; LDL, low-density lipoprotein; QUICKI, quantitative insulin sensitivity check index. *P < 0.05 vs. *quartile 1*; †*P* < 0.05 vs. *quartile 2*.

### Heart Rate in the Study Quartiles

**Figure 1.**
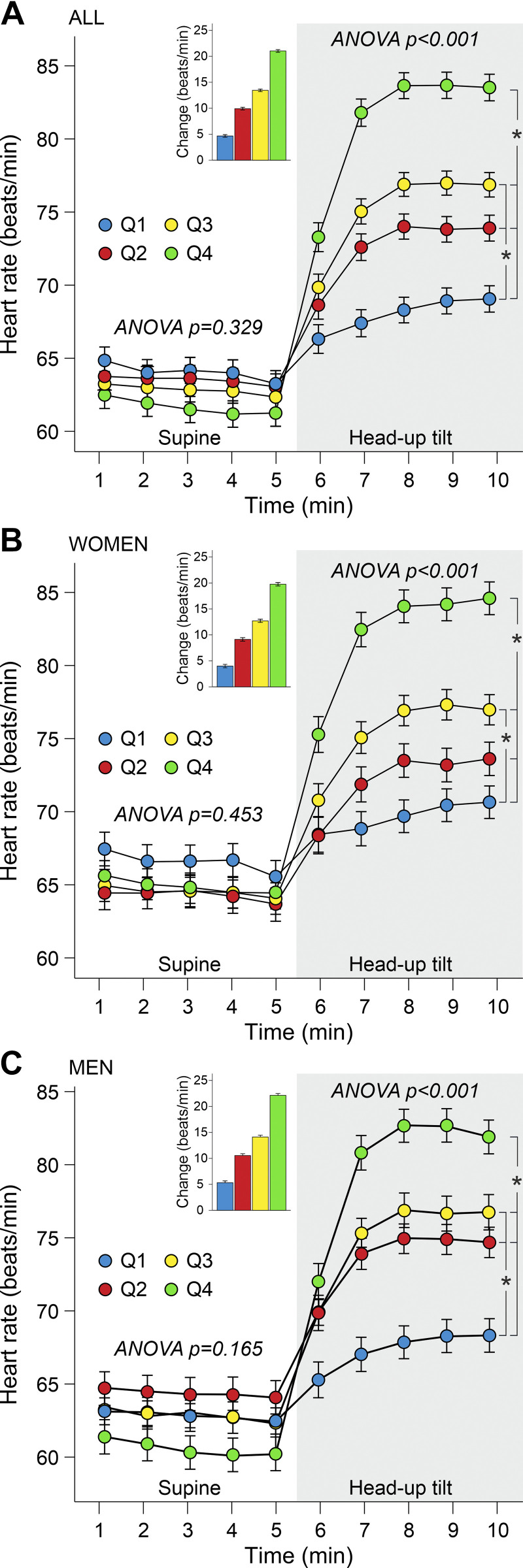
Heart rate in all participants (*A*), in women (*B*), and in men (*C*) during supine position and passive head-up tilt. Division to sex-stratified quartiles (*Q1*–*Q4*) was performed according to supine-to-upright change in heart rate; adjusted for age, body mass index, and fasting plasma glucose. The inserts show the supine-to-upright increases in heart rate in individual quartiles. Statistics are from the 5-min supine posture and last 3-min upright posture when the variables had stabilized, means (SE); *P < 0.05.

While in supine position, mean HR ranged between 60 and 65 beats/min, with no significant differences between the quartiles (Fig. 1*A*). During HUT, HR increased in all quartiles with lowest change in *Q1* (4.7 beats/min), moderate change in *Q2* and *Q3* (9.9 and 13.5 beats/min, respectively), and highest change in *Q4* (21.0 beats/min). The increases in HR were different in all quartiles (*P* < 0.001).

HR at rest was higher in women than in men (64.7 vs. 62.5 beats/min, *P* = 0.008), but during HUT, the attained HR was similar in both sexes (76.1 vs. 75.6 beats/min, *P* = 0.549). The increases in HR in the quartiles during HUT were 4.0, 9.2, 12.8, and 19.9 beats/min in women (Fig. 1*B*) and 5.3, 10.6, 14.2, and 22.2 beats/min in men (Fig. 1*C*). In all quartiles, the increases in HR during HUT were 1.3–2.3 beats/min lower in women than in the respective quartiles in men (*P* < 0.02 for all comparisons).

### Heart Rate Variability

**Figure 2.**
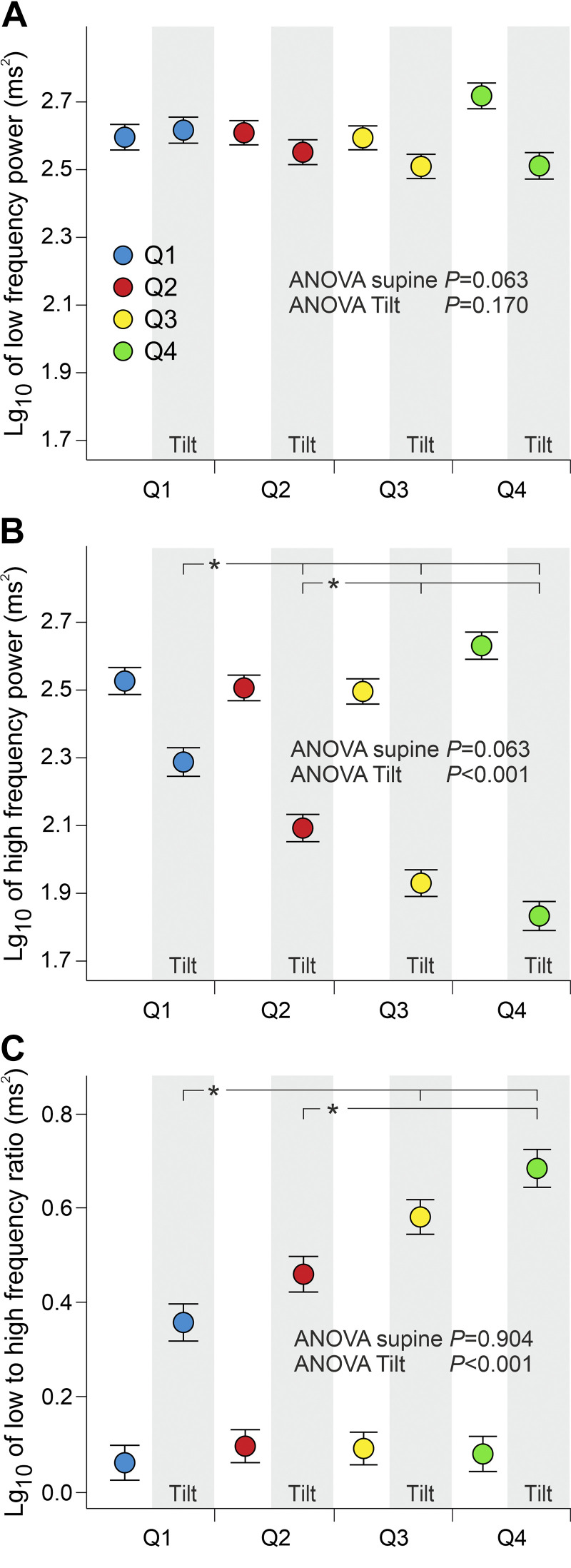
Power of heart rate variability (HRV) in low-frequency (*A*) and high-frequency (*B*) range, and low- to high-frequency ratio of HRV (*C*) during supine position and passive head-up tilt; analyses adjusted for age, body mass index, and fasting plasma glucose. Both sexes were divided separately to quartiles (*Q1*–*Q4*) according to supine-to-upright change in heart rate, lg_10_-transformed values are presented. White background (supine), gray background (upright); means (SE); *n* = 121 in *Q1*, *n* = 130 in *Q2*, *n* = 136 in *Q3*, and *n* = 120 in *Q4* (all participants did not have available HRV values); *P < 0.05.

Neither supine nor upright HRV values in the LF range were significantly different between the quartiles (Fig. 2*A*). Supine HRV in the HF range was also similar in all quartiles (Fig. 2*B*). Upright HRV in the HF range was highest in *Q1* and was also higher in *Q2* than in *Q3* and *Q4*. The LF:HF ratio of HRV, a variable that has usually been considered to mirror sympathovagal balance in the modulation of HR (27), was similar in all quartiles in the supine position. However, during HUT, the LF:HF ratio was lower in *Q1* than in *Q3* and *Q4* and lower in *Q2* than in *Q4* (Fig. 2*C*).

When women were compared with men, HRV in the LF range at rest did not differ between sexes but was lower during HUT in women. HRV in the HF range was always higher in women, and LF:HF ratio of HRV was always lower in women than in men (Supplemental Fig. S1; Supplemental Data may be found at https://doi.org/10.6084/m9.figshare.24798582). The sex-stratified HRV results are presented in the supplemental file (Supplemental Fig. S2). In both sexes, the differences in HR responses to upright posture were associated with variations in the HF power of HRV. However, no significant differences were observed in the LF power of HRV during upright posture when the quartiles of women and men were examined separately (Supplemental Fig. S2).

### Aortic Blood Pressure, Cardiac Variables, and Systemic Vascular Resistance

**Figure 3.**
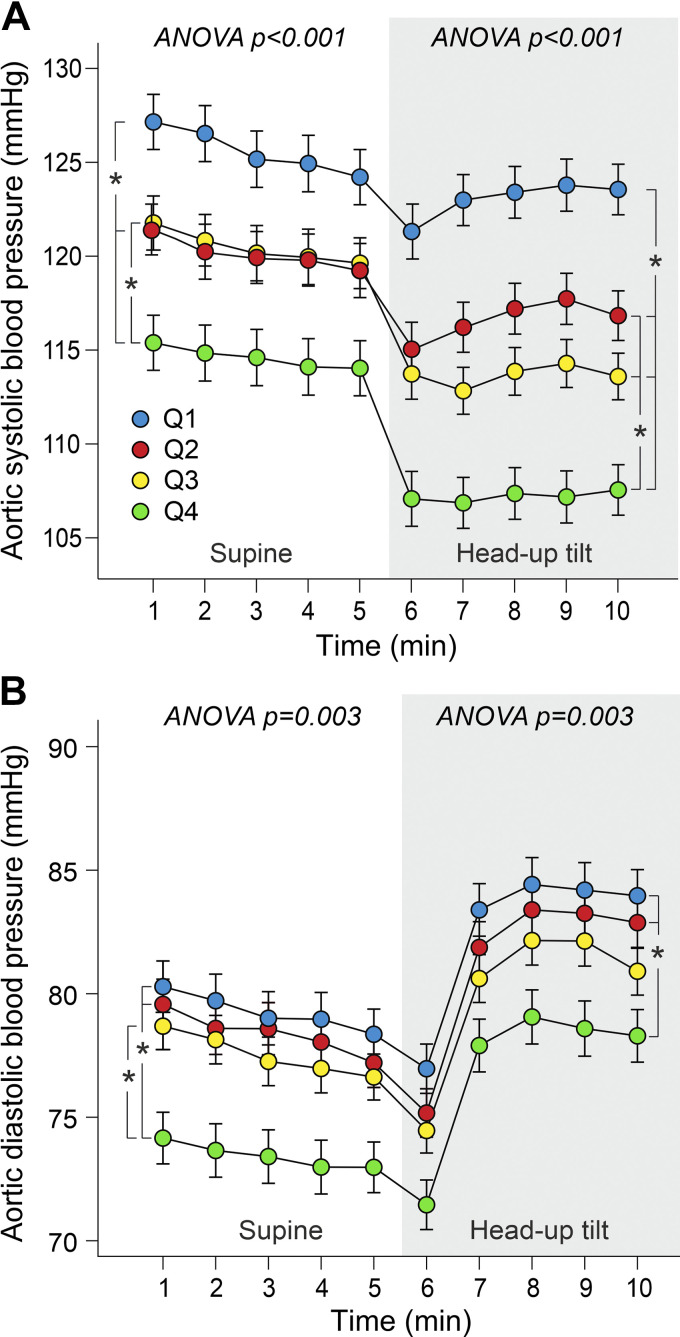
Aortic systolic blood pressure (*A*) and diastolic blood pressure (*B*) during supine position and passive head-up tilt. Both sexes were divided separately to quartiles (*Q1*–*Q4*) according to supine-to-upright change in heart rate; adjusted for age, body mass index, and fasting plasma glucose. Statistics are from the 5-min supine posture and last 3-min upright posture when the variables had stabilized, means (SE); *P < 0.05.

In supine position, mean aortic systolic BP was higher in *Q1* than in *Q2* and *Q4* and lower in *Q4* than in *Q1* and *Q3*. During HUT, mean aortic systolic BP was highest in *Q1* and lowest in *Q4*. Whether in supine or upright position, *Q2* and *Q3* presented with intermediate aortic systolic BP values (Fig. 3*A*). Supine aortic diastolic BP was lowest in *Q4*, whereas there were no differences between the other quartiles. During HUT, aortic diastolic BP was lower in *Q4* than in *Q1* and *Q2*, whereas the values did not differ between *Q1*, *Q2*, and *Q3* or between *Q3* and *Q4* (Fig. 3*B*).

**Figure 4.**
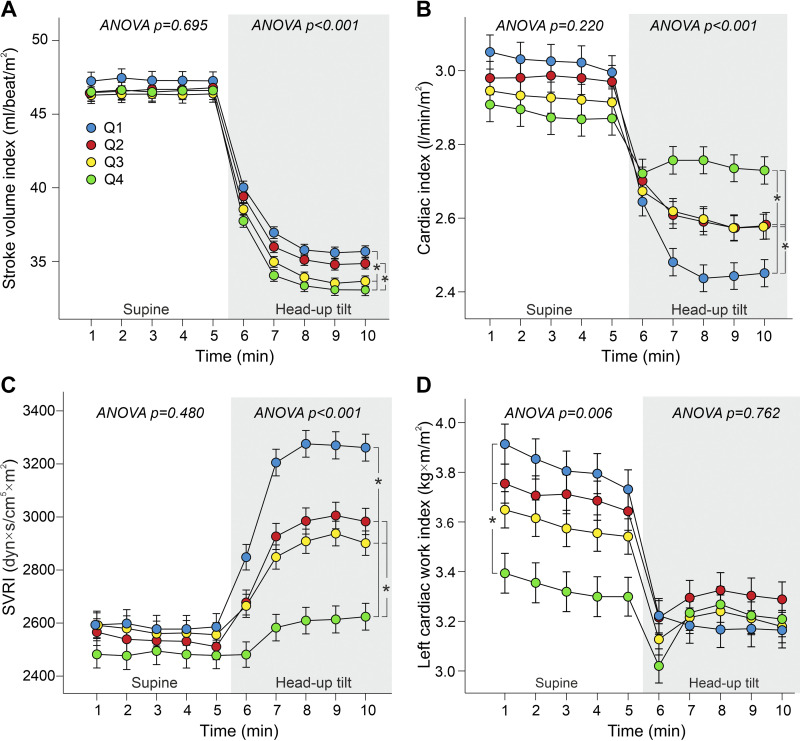
Stroke volume index (*A*), cardiac index (*B*), systemic vascular resistance index (SVRI) (*C*), and left cardiac work index (*D*) during supine position and passive head-up tilt, analyses are adjusted for age and fasting plasma glucose. Both sexes were divided separately to quartiles (*Q1*–*Q4*) according to supine-to-upright change in heart rate. Statistics are from the 5-min supine posture and last 3-min upright posture when the variables had stabilized, means (SE); *P < 0.05.

Supine stroke index (SI) values were similar in all quartiles (*P* = 0.695) and SI decreased in response to HUT (Fig. 4*A*). Although the differences during HUT were small, SI was higher in *Q1* than in *Q3* and *Q4*, and in *Q2* than in *Q4*. There were no significant differences in cardiac index (CI) values between the quartiles while in supine posture (Fig. 4*B*), but when in upright posture, *Q4* had the highest CI and *Q1* had the lowest CI, whereas *Q2* and *Q3* presented with intermediate values.

All quartiles had similar systemic vascular resistance index (SVRI) when in supine position (*P* = 0.480) (Fig. 4*C*), whereas the increase in SVRI during HUT was highest in *Q1* and lowest in *Q4* (650 vs. 173 dyn·s/cm^5^·m^2^, *P* < 0.001). Subsequently, upright SVRI remained lowest in *Q4*, and highest values were observed in *Q1*. Left cardiac work index (LCWI), a variable derived from mean aortic pressure and CI (22, 24), was higher in supine position in *Q1* and *Q2* than in *Q4*. Despite differences in BP (Fig. 3, *A* and *B*), upright LCWI was similar in all quartiles (Fig. 4*D*).

### Reproducibility of the Heart Rate Response During Head-Up Tilt

**Figure 5.**
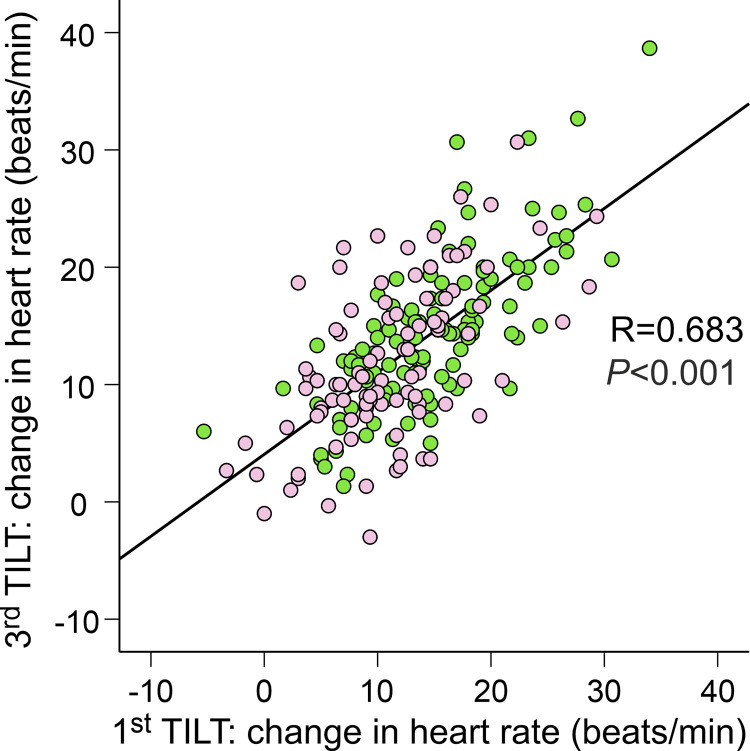
Scatter plot and Pearson correlation of the heart rate changes during the first and the third passive head-up tilt from supine to upright position performed 90 days (median) apart in 215 subjects; women depicted with pink symbols and men depicted with light green symbols.

Among the 215 subjects in the reproducibility analyses, women and men were also divided separately to sex-specific quartiles according to the HR response during the first HUT (Table 3). These participants were subjected to three HUTs so that the median difference between the first and the second challenge was 61 days, and the median difference between the second and the third challenge was 29 days. The phenotype of the HR change was reproducible as the average heart rate increases in all 215 subjects during the first, second, and third HUTs were 13.1 (6.6), 13.6 (7.1), and 13.1 (6.8) beats/min, respectively [means (SD), *P* = 0.681]. In *Q1*, the heart rate response to the first HUT was lower than during the second and third HUTs, but in the other quartiles, the repeated heart rate responses to HUT did not differ (Table 3). Importantly, the increase in HR during HUT was always lowest in *Q1* and highest in *Q4*. The change in HR during HUT was highly correlated between the first and the second (*r*_P_ = 0.616, *P* < 0.001), the second and the third (*r*_P_ = 0.674, *P* < 0.001), and the first and the third challenges (*r*_P_ = 0.683 for all participants, *r*_P_ = 0.579 for women, *r*_P_ = 0.739 for men, *P* < 0.001 for all) (Fig. 5).

**Table 3.** Increases in heart rate during 3 passive HUTs on separate days in the quartiles of the supine-to-upright change in heart rate divided separately for sexes

	*Quartile 1*	*Quartile 2*	*Quartile 3*	*Quartile 4*	ANOVA, *P* Value
*n*	54	51	60	50	
Change in heart rate					
1st HUT	5.4 (3.3)	10.6 (2.2)*	14.7 (2.4)*†	21.5 (4.6)*†‡	<0.001
2nd HUT	8.8 (6.4)§	12.4 (4.4)*	13.8 (5.8)*	20.0 (6.6)*†‡	<0.001
3rd HUT	8.2 (4.6)§	11.1 (4.6)*	13.9 (6.0)*†	19.5 (6.1)*†‡	<0.001

Values are means (SD); *n*, number of participants. Median time difference was 61 days between 1st and 2nd challenge and 29 days between 2nd and 3rd challenge. *P < 0.05 vs. *quartile 1*; †*P* < 0.05 vs. *quartile 2*; ‡*P* < 0.05 vs. *quartile 3*; §*P* < 0.05 vs. 1st head-up tilt (HUT).

## DISCUSSION

Our study discovered reproducible changes in HR responses during passive HUT. The phenotype with the lowest increase in HR (5 beats/min) had numerically highest aortic systolic BP before and after HUT, in addition to lowest cardiac output and highest systemic vascular resistance during HUT. The phenotype with the highest increase in HR (21 beats/min) had numerically lowest systolic and diastolic BP regardless of posture, as well as highest cardiac output and lowest systemic vascular resistance during HUT. The HRV results indicated underlying differences in the parasympathetic modulation of upright HR, with highest HF power of HRV in the quartile with lowest increase in HR and lowest HF power of HRV in the quartile with highest increase in HR. Although HRV results indicated stronger parasympathetic modulation of heart rate in women than in men, the differences in the HR responses to upright posture were associated with variations in the HF power of HRV in both sexes. These findings emphasize the importance of vagal regulation underlying the differences in posture-related changes in HR. Despite the phenotypic differences, upright left cardiac work was similar in all groups.

The mean BMI in the DYNAMIC study cohort was 26.5 kg/m^2^, which well corresponds to the Finnish population, as in a recent survey the mean BMI was 27.7 kg/m^2^ in men and 27.5 kg/m^2^ in women (31). Importantly, 98% of the present subjects were free from medications interfering with cardiovascular function. The relatively large number of DYNAMIC participants (*n* = 574) included normotensive and never-treated hypertensive subjects. All participants were of Finnish descent, and detailed information on demographic and laboratory characteristics was given. The subjects with the largest increase in HR (*Q4*) during HUT were youngest, had lower BMI than those in *Q2*, and lowest aortic BP regardless of posture apart from *Q3* during HUT. Fasting plasma glucose was 0.25 mmol/L lower in *Q4* than in *Q1*, whereas other laboratory results were similar in the quartiles. After adjusting for age, the quartiles only differed in BMI and fasting plasma glucose concentrations, and the comparisons were adjusted for these differences. Mean plasma glucose was also in the normal range in all quartiles. Thus, the small deviations in glucose concentrations between *Q4* and *Q1* cannot explain the differences in the HR responses during HUT. Based on analyses of PWV, no differences in large arterial stiffness were observed between the quartiles.

HR is an indicator of life span (32), while Levine (33) suggested that mammals of all sizes, including humans, have on average ∼10 × 10^8^ heart beats per lifetime, despite different life expectancies. Increased resting HR is a risk factor for elevated BP in children and adolescents, regardless of age, ethnicity, or adiposity (34). Higher resting HR has been associated with clinical features of the metabolic syndrome (29, 35), and it is a predictor of coronary heart disease (6) and heart failure (7). Also, lower increase in HR during exercise and prolonged HR recovery after exercise are associated with mortality (36). The present findings do not dispute the fact that several lines of previous evidence have linked elevated resting HR with cardiovascular morbidity and mortality (5–8). However, HR during upright posture cannot be reliably predicted from HR at rest, and routine measurements of HR and BP in supine, seated, and standing postures could improve cardiovascular risk stratification (2, 3, 37, 38). Modern oscillometric BP recording devices can provide additional information about hemodynamics, including wave reflection, arterial stiffness, cardiac output, and peripheral arterial resistance (37, 39), taking the description of the cardiovascular phenotype much further than with the original method described by Korotkoff in 1905 (40).

The mechanisms by which a higher resting heart rate increases cardiovascular risk are not properly understood. Sympathetic overactivity is a putative link between elevated HR and cardiovascular events (11, 12). Higher HR at rest has been related to elevated sympathetic influence on cardiac autonomic modulation (10) and to a more hyperdynamic hemodynamic profile, whether in supine or upright position (9). Additional factors associating higher resting HR with less favorable prognosis are higher cardiac workload and increased arterial stiffness (9, 11). Elevated BP may be one of the most potential mechanisms associating higher HR with cardiovascular risk (41). Previous studies have pondered on the lack of a certain HR threshold, under which the risks would level off and over which the risks would increase (5, 41). The phenotypic variability in the supine-to-upright change in HR potentially explains why such a threshold has not been uncovered. Based on the present results, HR at rest alone is not a reliable means to classify subjects to cardiovascular phenotypes. Of note, the phenotype with the highest increase in HR during HUT did not have hyperdynamic circulation at rest.

HRV is a noninvasive method used to assess autonomic nervous system effects on heart rate (42). Reduced HRV has been previously reported to predict cardiovascular diseases (6) and heart failure (7). Parasympathetic and sympathetic modulation of HR have been evaluated using time- and frequency domain HRV assessments (27). The present study examined the frequency domain variables, with the HF component reflecting vagal HR control according to broad previous evidence (42–45). During periods of low levels of physical stress, heart rate is mainly subject to parasympathetic control (43), and the stress induced by passive HUT can be expected to fall into this category.

LF power of HRV provides insights into cardiac autonomic activity, but the results must be interpreted with caution. Both sympathetic and parasympathetic activity influence LF power, whereby it is an imperfect measure of cardiac autonomic balance (42, 43). There is individual variation in the relationship between LF power and sympathetic activity (43), while changes in respiration and thermoregulation can also affect LF power without parallel changes in sympathetic activity (42, 43). The LF power of HRV has even been reported to more reflect modulation of cardiac autonomic outflow by baroreflexes than by sympathetic tone (44).

In the analysis where all participants of the present study were included, we found no significant differences in the LF power between the quartiles whether in supine or upright position and no HF power differences among the quartiles in the supine position. However, higher HR during passive HUT was related with lower HF power, with differences between all other quartiles except for *Q3* and *Q4*. Thus, higher increase in HR during HUT was explained by lower modulation of HR in the HF range, which reflects the parasympathetic component of HR control (42–45). Although studies on cardiovascular pathophysiology have frequently centered around sympathetic overactivity, differences in parasympathetic tone may play a significant role in the cardiovascular risk associated with an imbalance in autonomic tone (22, 46, 47).

Cardiovascular status is usually assessed in the seated posture (48), but this approach excludes information about posture-related differences in hemodynamics, which exhibit considerable variability (2, 15, 22). Recent evidence indicates that cardiovascular status should be evaluated both at rest and during upright posture. In the Systolic Blood Pressure Intervention Trial (SPRINT) study, ∼21% of the participants exhibited orthostatic hypertension at baseline (defined as a ≥20-mmHg increase in systolic BP or ≥10-mmHg increase in diastolic BP), which was associated with a 1.44-fold hazard ratio for developing cardiovascular outcomes when compared with participants without orthostatic hypertension (38). Over a 17-year follow-up period, an exaggerated systolic BP response and a higher increase in HR upon standing predicted adverse cardiovascular events among 1,207 Italian subjects (3). In the present study, the different phenotypes of the supine-to-upright change in HR presented marked underlying differences in the regulation of cardiac output and systemic vascular resistance, and such findings have potential clinical significance. Upright cardiac output remained highest in the quartile with highest supine-to-upright increase in HR and lowest BP, suggesting that sufficient cardiac output was required for sustaining BP when in upright position. In contrast, upright systemic vascular resistance was highest in the quartile with lowest upright HR. Although upright aortic systolic BP was highest in *Q1* and lowest in *Q4* and upright aortic diastolic BP was lower in *Q4* than in *Q1* and *Q2*, left cardiac work during HUT was similar in all quartiles. This was an unexpected finding and shows that all quartiles were at a comparable level of cardiovascular load before possible upcoming physical activity that takes place in the upright posture.

The supine-to-upright change in HR was a reproducible phenotype during 90 days of follow-up among 215 participants, who were examined in quartiles each containing 51–60 subjects. The quartiles retained their characteristic HR increase during successive HUTs performed 61 and 29 days (medians) apart. Furthermore, the change in HR in response to HUT was highly correlated between the first and the third challenges. Therefore, the HR responses during HUT were highly reproducible on separate occasions.

The limitations of the present study should be acknowledged. We applied noninvasive methods, and aortic BP was mathematically derived from the radial artery tonometric signal (23). PWV was evaluated from the time difference between sequential impedance changes in the whole body and popliteal region, whereas evaluations of stroke volume and cardiac output were derived from the bioimpedance signal (22, 25, 26). Nevertheless, these methods have been validated by comparing the results to invasive measurements, three-dimensional ultrasound, and tonometric PWV recordings (9, 25, 26, 49). The present recordings lasted for 10 min, which is a relatively short time window for examining hemodynamics. However, when compared with single measurements of BP and HR, on average, the present calculations were based on data from over 700 cardiac cycles in the study subjects. Criticism regarding the reliability of tonometric BP recordings has been published (50, 51). Yet, we recently found that the tonometric BP values closely matched the ambulatory daytime BP in over 410 subjects (52). Changes in the hormonal profile during the menstrual cycle may also have affected the HRV results in the female participants (53).

To conclude, our results indicate that the change in HR during HUT is a reproducible hemodynamic phenotype with underlying differences in the modulation of cardiac parasympathetic tone and regulation of systemic vascular resistance. As the HR responses to HUT are not uniform, resting HR obtained in the supine position alone is not a reliable means to classify subjects to cardiovascular phenotypes. Future research should clarify the prognostic significance of the phenotypic differences.

## DATA AVAILABILITY

Analyses and generated data sets that support the current study are not available publicly. The data sets are available from the corresponding author on reasonable request.

## SUPPLEMENTAL DATA

10.6084/m9.figshare.24798582 Supplemental Figs. S1 and S2: https://doi.org/10.6084/m9.figshare.24798582.

## GRANTS

This work was supported by Finnish Foundation for Cardiovascular Research (to I.H.P.), Sigrid Jusélius Foundation (to I.H.P.), Competitive State Research Financing of the Expert Responsibility Area of Tampere University Hospital Grants 9AB057 and 9AC076 (to I.H.P.), Päivikki and Sakari Sohlberg Foundation (to I.H.P.), Pirkanmaa Regional Fund of the Finnish Cultural Foundation (to H.B. and I.H.P.), and the city of Tampere (to H.B.).

## DISCLOSURES

No conflicts of interest, financial or otherwise, are declared by the authors.

## AUTHOR CONTRIBUTIONS

H.B. and I.H.P. conceived and designed research; J.K.K., A.T., M.H., and I.H.P. performed experiments; H.B. and I.H.P. analyzed data; H.B. and I.H.P. interpreted results of experiments; H.B. and I.H.P. prepared figures; H.B. and I.H.P. drafted manuscript; H.B., J.K.K., A.T., M.H., T.P.H., J.T.M., and I.H.P. edited and revised manuscript; H.B., J.K.K., A.T., M.H., T.P.H., J.T.M., and I.H.P. approved final version of manuscript.
